# Interventions to Address Clinical Incivility in Nursing: A Systematic Review

**DOI:** 10.3390/nursrep15060199

**Published:** 2025-06-03

**Authors:** Anne Lama, Henrietta Nwamu, Younglee Kim

**Affiliations:** Department of Nursing, California State University San Bernardino, 5500 University Parkway, San Bernardino, CA 92407, USA; alama@csusb.edu (A.L.); henrietta.nwamu@csusb.edu (H.N.)

**Keywords:** clinical incivility, intervention, nursing education, practice

## Abstract

**Background/Objectives:** Clinical incivility is a persistent issue in nursing education and practice, with negative impacts on students, educators, and clinicians. Uncivil behaviors—such as belittling, exclusion, and dismissiveness—compromise communication, teamwork, and patient safety. Although various interventions have been implemented, their effectiveness remains inconsistent. This systematic review aimed to evaluate the effectiveness of interventions addressing clinical incivility in nursing and to identify common trends, gaps, and implications for future practice and research. **Methods:** Following PRISMA 2020 guidelines, a systematic search was conducted in PubMed, Web of Science, and EBSCOhost (CINAHL) for peer-reviewed empirical studies published between 2014 and 2024. Search terms included “clinical incivility” and (“intervention” or “program” or “training”) and “nursing”. Studies were eligible if they evaluated interventions aimed at reducing incivility among nursing students, faculty, or practicing nurses. Seventeen studies met the inclusion criteria and were analyzed for intervention types, target populations, delivery methods, and outcomes. **Results:** The review identified five main intervention types: educational modules (n = 9), cognitive rehearsal (n = 5), simulation and role-play (n = 5), team-based strategies (n = 3), and feedback/communication strategies (n = 2). Many studies used multiple strategies. Fourteen studies reported positive outcomes such as improved awareness, communication, and self-efficacy. Eight studies demonstrated statistically significant reductions in perceived incivility, particularly those with simulation-based, multi-session, or institutionally supported formats. Three studies showed limited or mixed results due to insufficient follow-up or lack of leadership engagement. **Conclusions:** Experiential and multi-component interventions appear effective in reducing clinical incivility. Long-term success requires leadership engagement, institutional support, and integration into ongoing professional development.

## 1. Introduction

Worldwide, an abundance of uncivil experiences in nursing have been documented with resultant negative repercussions, yet the progression of “zero tolerance” has been inconsistent, moving at a snail’s pace [1]. As a profession, nursing is grounded in a standard of conduct based on civility, which is foundational to professional nursing practice and critical for fostering safe, respectful, and high-functioning healthcare environments. Civility promotes collaboration, psychological safety, and optimal patient outcomes. The 2021 American Association of Colleges of Nursing (AACN) Essentials: Core Competencies for Professional Nursing Education, although not explicit, embeds the concept of civility as an essential component of competency-based education throughout, especially in the domains of person-centered care, interprofessional partnerships, and professionalism [2]. In contrast, incivility—defined as low-intensity, disrespectful behaviors such as belittling, exclusion, eye-rolling, and dismissiveness—undermines trust, disrupts communication, and compromises both team dynamics and patient care [3,4]. These behaviors are often subtle but cumulative in their impact, leading to emotional distress, job dissatisfaction, burnout, and increased turnover across nursing roles [5,6,7,8,9].

Identified in the literature since 1986 [10] and further addressed as still “eating our young” [11], hundreds of articles and research written on incivility and nursing continue today. Considered a nursing “rite of passage” [12], incivility has been explored in various ways and in several healthcare and academic environments, along with various ways to mitigate it, starting with interventions and educational programs in healthcare institutions throughout the world. Since 2011, The Institute of Medicine’s Initiative on the Future of Nursing, followed by the National Academies of Sciences, Engineering, and Medicine’s (NASEM) Future of Nursing 2020–2030, supports a key takeaway message between quality patient care and safety and nursing as critical to success [13,14]. However, when research links incivility to the compromise of a nurse’s primary focus of safe and quality patient care, interventions need to be put into play from day one of a nursing students’ program to tackle this chronic issue. The bottom line is that the strategy of zero tolerance and priority of a culture of respect to tackling bullying behavior addressed in the American Nurses Association’s (ANA) position statement [15] is falling on deaf ears. Tools have been created and validated to measure the perceptions of incivility toward nursing students [16], as well as the repercussions of bullying on nursing students’ self-esteem [17,18]. However, current global research and reviews further find continued persistence rather than actual reduction of incivility in nursing [19,20,21], to the point of being called an “epidemic” [22].

Clinical incivility is not limited to nursing student experiences in clinical education; it is a widespread and persistent problem that affects nurses at all levels—from novice students to seasoned practitioners, educators, and leaders—across both academic and healthcare settings. Studies have shown that nursing students often encounter incivility during clinical placements, while practicing nurses frequently face similar challenges in their work environments, leading to emotional distress and professional disengagement [6,23,24,25]. The consequences are far-reaching: incivility contributes to nurse attrition, toxic workplace cultures, reduced quality of care, and diminished organizational performance [26,27]. With the growing strain on the global nursing workforce, exacerbated by burnout and staffing shortages, addressing incivility is a matter of urgency for sustaining the profession and ensuring high-quality, compassionate care. Recent qualitative research by Roberts [28] emphasized how incivility in clinical placements leads student nurses to feel disrespected, intimidated, and professionally undermined, ultimately disrupting learning and identity development.

Despite increased awareness and calls for action, the nursing discipline lacks a cohesive, evidence-based approach to managing and reducing clinical incivility [11,29,30]. Numerous interventions have been introduced, ranging from communication and conflict resolution training to system-wide cultural transformation initiatives. However, existing research is fragmented, often context-specific, and varies in terms of design, implementation, and evaluation [25]. Without a clear understanding of what works, for whom, and under what circumstances, efforts to reduce incivility risk being ineffective or unsustainable [31]. Attempts are needed to develop and identify appropriate and effective methods. Based on a clear understanding of what interventions have been attempted so far, it will be necessary to develop or evaluate tailored interventions that are both effective and meaningful.

This systematic review addresses a critical gap in the literature by synthesizing the last decade of research on interventions to reduce clinical incivility across the nursing discipline [20,31]. By evaluating the scope, design, and effectiveness of these interventions, this review aims to provide clarity on best practices, highlight areas for improvement, and inform future strategies. The findings are intended to guide educators, administrators, policymakers, and frontline clinicians in adopting evidence-informed solutions to foster a culture of civility and respect within nursing. In doing so, this review contributes not only to advancing academic understanding of incivility interventions but also to shaping practical, scalable responses that support workforce resilience, professional well-being, and safer healthcare systems.

## 2. Methods

### 2.1. Aim

This systematic review aimed to evaluate the effectiveness of interventions, programs, or training sessions designed to address clinical incivility in nursing over the past ten years. Specifically, this review sought to achieve the following:

(a) Identify evidence-based strategies implemented in nursing education and clinical settings to address and mitigate clinical incivility among nursing students, faculty, and practicing nurses.
(b) Evaluate the impact of these interventions on the well-being of nursing students, nurse educators, and practicing nurses.
(c) Determine their effectiveness in fostering a respectful learning and working environment.
(d) Explore trends in intervention approaches and identify gaps in the existing literature.
(e) Provide recommendations for future policies and practices to promote a culture of civility in nursing.

### 2.2. Design

This study followed the Preferred Reporting Items for Systematic Reviews and Meta-Analyses (PRISMA) 2020 guidelines, ensuring a transparent, rigorous, and reproducible approach to identifying, screening, and selecting studies for review. The PRISMA checklist was applied to enhance the reliability and validity of the systematic review methodology.

### 2.3. Eligibility Criteria

The eligibility criteria for study selection were established based on the population, intervention, comparison, outcome, and study design (PICOS) framework.

#### 2.3.1. Inclusion Criteria

Studies were included if they met the following criteria:

- Focused on nursing students, faculty, or practicing nurses who experienced uncivil behaviors or language in academic or clinical environments.
- Published between January 2014 and December 2024.
- Peer-reviewed and written in English.
- Examined at least one intervention, program, or training session designed to address clinical incivility in nursing education or practice.
- Utilized a qualitative, quantitative, or mixed-method research design to evaluate intervention outcomes.
- Included interpersonal incivility occurring between nursing staff members, among nursing students, between healthcare staff and nursing students, between patients’ families and nursing staff or students, between clinical faculty and students, and between nursing staff and students in clinical settings.

#### 2.3.2. Exclusion Criteria

Studies were excluded if they met any of the following conditions:

- Not written in English.
- Published outside the timeframe of January 2014 to December 2024.
- Did not focus on clinical incivility experienced by nursing students, faculty, or nurses.
- Did not include an intervention, program, or training session addressing clinical incivility.
- Did not employ an empirical research design (qualitative, quantitative, or mixed-methods).
- Focused on incivility directed toward patients rather than that experienced by nursing students, faculty, or nurses.

### 2.4. Information Sources and Search Strategy

A systematic literature search was conducted across three electronic databases to identify relevant studies: (a) EBSCOhost Academic Premier, including CINAHL, (b) Web of Science (WOS), and (c) PubMed.

Each of the three authors independently searched an assigned database using the primary keywords, which included Clinical Incivility and Interventions or Programs or Training and Nursing. To enhance the relevance and quality of retrieved studies, the following filters were applied:

- Peer-reviewed publications only—ensuring that only studies that had undergone expert review were included.
- Publication time frame (January 2014–December 2024)—restricting results to studies published within this period.
- English-language studies—removing articles published in other languages.
- Empirical research only—excluding opinion pieces, editorials, and conference abstracts.
- After applying these filters, the final search was completed in February 2025, and the retrieved references were prepared for screening.

### 2.5. Study Selection and Screening

A structured, multi-step screening process was implemented to ensure the inclusion of only relevant and eligible studies:

(1) Title and Abstract Screening—all authors independently reviewed the titles and abstracts of retrieved articles to exclude those that were clearly irrelevant.
(2) Full-Text Review—articles that passed the initial screening were assessed in full to confirm they met the predetermined inclusion and exclusion criteria.
(3) Manual Duplicate Removal—since records were retrieved from multiple databases (EBSCOhost, Web of Science [WOS], and PubMed), manual de-duplication was performed. Duplicates were identified and removed by comparing study titles, authors, publication years, and journal information.
(4) Consensus Resolution—any disagreements regarding inclusion or exclusion were resolved through collaborative discussion among the authors until consensus was reached.

#### Screening Outcomes

A total of 180 articles were identified through database searching:

- EBSCOhost (including CINAHL): 106
- Web of Science (WOS): 63
- PubMed: 11

Following title, abstract, and full-text screening, 146 articles were excluded for the following reasons:

- Lack of an intervention, program, or training component: EBSCOhost (75), WOS (41), and PubMed (3) (n = 119 excluded).
- Lack of relevance to the study focus: EBSCOhost (16), WOS (8), and PubMed (1) (n = 25 excluded).
- Language restrictions: EBSCOhost (2) (n = 2 excluded)

This resulted in 33 articles retained for full-text review: EBSCOhost (13), WOS (13), and PubMed (7). After manual review and duplicate removal, a total of 17 unique studies were included. Thirteen duplicate articles appeared across EBSCOhost (13), WOS (10), and PubMed (6). Additionally, 3 unique studies from WOS and 1 from PubMed were retained.

### 2.6. Data Collection Process

A structured data extraction form was used to systematically extract information from the selected studies. All three authors independent reviewers collected and verified data, extracting the following details:

- Study characteristics (authors, publication year, and country).
- Study design and methodology (qualitative, quantitative, or mixed-methods).
- Intervention details (type, duration, and target population).
- Outcome measures (impact on incivility, student/faculty/nurse perceptions, and effectiveness).

### 2.7. Risk of Bias Assessment Methodology

To evaluate the methodological quality and potential bias within the 17 included studies, an informal appraisal approach was used. Rather than applying standardized tools, the review team relied on established bias domains to guide critical judgment and promote consistency across study evaluation. This approach was deemed appropriate given the diversity in study design, which included qualitative, mixed-methods, quasi-experimental, and pilot studies.

The following domains were considered for each study:

- Selection Bias—how participants were recruited and whether the sampling method could introduce bias.
- Performance Bias—the potential influence of awareness of group allocation or intervention exposure on behaviors or outcomes.
- Detection Bias—the clarity, objectivity, and consistency of outcome measurement and evaluation.
- Reporting Bias—whether the study transparently reported outcomes, including negative or null findings.

Each author reviewed the studies independently, and assessments were compared and discussed during team meetings. Any discrepancies in judgments were resolved through consensus. Although informal, this structured review process helped ensure a consistent appraisal of methodological strengths and limitations across the evidence base.

### 2.8. Data Synthesis and Analysis

The findings from the included studies were systematically synthesized. The synthesis process involved the following:

(1) Tabulating study characteristics and intervention details.
(2) Identifying common themes, intervention strategies, and study findings.
(3) Assessing the effectiveness of interventions using outcome measures.
(4) Evaluating the impact of interventions on nursing students, faculty, and nurses.

## 3. Results

**Figure 1:**
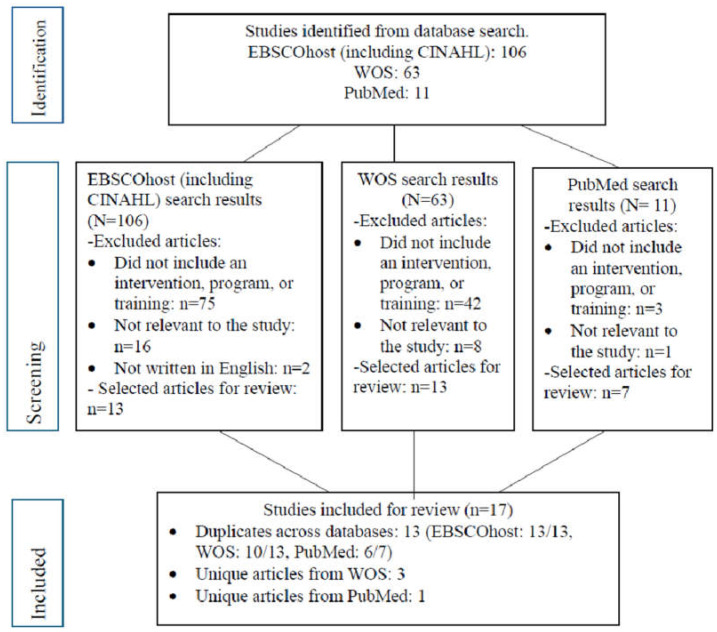
PRISMA flow diagram of studies included for review.

A total of 17 studies met the inclusion criteria and were included in this systematic review. The selection process is illustrated in the PRISMA flow diagram (see Figure 1). The included studies were published between 2014 and 2024 and conducted across four countries: the United States (n = 13), Iran (n = 2), South Korea (n = 1), and Taiwan (n = 1).

### 3.1. Study Characteristics

Most studies employed quasi-experimental, pilot, or pre–post-intervention designs (n = 8), while others used mixed-methods approaches (n = 5), randomized controlled trials (n = 2), or qualitative designs (n = 2). Sample sizes varied widely, ranging from as few as 11 to more than 1500 participants. The studies targeted diverse populations, including undergraduate nursing students, newly licensed registered nurses, and clinical and academic nursing staff. Detailed study characteristics—such as authorship, country, sample population, intervention type, study design, and key findings—are presented in Table 1.

### 3.2. Prevalence of Clinical Incivility in Nursing

Across the reviewed literature, the prevalence of incivility varied depending on the study population, clinical setting, and measurement instruments. Among pre-licensure nursing students and practicing nurses, reported rates of exposure to incivility or bullying behaviors ranged from 25.5% to over 85%, indicating a widespread and persistent issue in both educational and professional environments.

In clinical learning environments, several studies highlighted high levels of incivility experienced by nursing students. Kim et al. [24] reported that 71.4% of senior BSN students in the United States experienced incivility from registered nurses, while Kim et al. [29] found a slightly higher rate of 72.73% among Korean BSN students, with incidents primarily involving disrespect and exclusion by clinical nurses. Similarly, Tecza et al. [16] observed substantial levels of incivility directed toward nursing students during clinical rotations, though a specific prevalence rate was not reported. Additionally, Rose et al. [32] cited prior research indicating that 70% of nursing students experienced peer incivility in academic settings.

A growing body of literature also documents the prevalence of horizontal incivility, or peer-to-peer mistreatment, particularly among newly graduated and novice nurses. McDermott et al. [33] reported that 29% to 57% of first-year nurses experienced incivility directly. Similarly, Garcia et al. [34] and Howard and Embree [35] identified workplace incivility as a significant contributor to new nurse turnover, underscoring its impact on retention during the early stages of practice. Aebersold and Schoville [36] found that 39% of new nurses experienced horizontal bullying from peers, with novice nurses more frequently targeted than experienced colleagues. Supporting these findings, Nikstaitis and Simko [37] and Tsai and Chou [38] reported that up to 85% of nurses had been exposed to horizontal hostility. Notably, they also found that incivility extended beyond nurse-to-nurse interactions, with participants reporting mistreatment from patients and their families as well.

### 3.3. Types of Clinical Incivility in Nursing

Verbal incivility includes rude or dismissive language, sarcastic remarks, and public criticism, often experienced by both students and nurses in clinical settings [24,33,37,39]. Non-verbal incivility is characterized by eye-rolling, sighing, ignoring, or excluding others, which communicates disrespect and can significantly undermine collaboration and learning [16,29,38]. Horizontal incivility refers to peer-to-peer mistreatment, such as gossiping, withholding information, undermining, or sabotaging coworkers, and is especially prevalent among newly graduated nurses [36,37,40]. Nursing students frequently encounter incivility from clinical staff such as nurses, including being ignored, dismissed, or treated as burdens, which negatively affects their confidence and learning [16,24,29,32]. Incivility from patients and families includes verbal abuse, unreasonable demands, and a lack of respect toward nurses, all of which contribute to emotional strain and professional burnout [37,38]. Additionally, organizational and structural incivility—such as poor communication, inconsistent feedback, inadequate faculty support, and the absence of formal civility policies or training—can foster a toxic learning and work environment [35,41,42].

### 3.4. Intervention Approaches

While many studies employed a single intervention modality, several incorporated multiple instructional components. Five studies integrated cognitive rehearsal with other teaching strategies. Clark et al. [43] combined cognitive rehearsal with simulation-based scenarios and debriefing using the TeamSTEPPS CUS model. Tsai and Chou [38] developed a mobile application that included cognitive rehearsal, education on incivility, interactive video scenarios, and role-play dialogue practice. Razzi and Bianchi [40] implemented cognitive rehearsal within a quality improvement program that included scripted responses, role-playing, and structured education. McDermott et al. [33] incorporated cognitive rehearsal into a nurse residency program through didactic instruction, role-play, cue cards, and practice with personalized scripts. Similarly, Kousha et al. [39] delivered cognitive rehearsal across five sessions, which included education on incivility, presentation of ten common uncivil scenarios, appropriate responses, and role-play. While the interventions varied in format and delivery, they were primarily classified into five key categories based on their core instructional approaches.

#### 3.4.1. Educational Modules and E-Learning (Nine Studies)

Structured educational programs were the most frequently used intervention type, applied across nine studies. These interventions ranged from brief single-session workshops to multi-week, interactive modules that aimed to increase awareness, improve communication, and build assertiveness in responding to incivility. Kim et al. [24] delivered a three-week incivility management program that incorporated video-based clinical scenarios depicting common uncivil encounters. These were followed by guided small-group discussions focused on identifying types of incivility, their consequences, and effective strategies for respectful communication. Abedini et al. [44] implemented a dialogue-driven training series grounded in professional civility, where participants engaged in reflective exercises and peer discussions to examine their own responses to incivility and practice giving constructive feedback. Howard and Embree [35] provided asynchronous e-learning for staff nurses, which included narrated case studies, interactive quizzes, and communication frameworks for addressing lateral violence. Nikstaitis and Simko [37] offered in-service training focused on recognizing subtle forms of incivility and applying scripted response techniques in clinical situations. Kim et al. [29] developed a two-hour session integrating small-group case-based discussions and debriefings, enabling participants to explore ethical and professional responses to disrespectful behaviors. Razzi and Bianchi [40] embedded targeted educational content—such as identifying behavioral triggers and practicing scripted responses—into a broader cognitive rehearsal framework. Altmiller et al. [41] implemented a civility education initiative across nine academic institutions, emphasizing constructive feedback techniques and respectful dialogue through video modules and guided feedback practice. Lastly, McDermott et al. [33] included a one-hour didactic lecture as part of a cognitive rehearsal intervention for newly licensed nurses, focusing on developing verbal scripts and improving communication clarity when facing incivility in real clinical environments.

#### 3.4.2. Cognitive Rehearsal Training (Five Studies)

Cognitive rehearsal was a central component in several interventions aimed at preparing nurses and nursing students to respond effectively to workplace incivility. Studies by McDermott et al. [33], Tsai and Chou [38], Kousha et al. [39], Razzi and Bianchi [40], and Clark et al. [43] implemented this approach through various formats, including live workshops, app-based simulations, scenario-based role-plays, and online interactive modules that promoted reflective dialogue and respectful communication. These interventions involved practicing scripted responses to common uncivil scenarios, enhancing participants’ assertiveness and perceived readiness. While most studies reported improvements in recognizing and addressing incivility, some found that cognitive rehearsal alone may be insufficient without continued support or reinforcement in real-world settings.

#### 3.4.3. Simulation and Role-Play (Five Studies)

Simulation-based learning was widely used across five studies to immerse participants in realistic, high-pressure scenarios involving workplace or academic incivility. Clark et al. [43] employed simulation exercises combined with biometric data collection (e.g., salivary stress biomarkers) to explore physiological responses to incivility during clinical handoffs. Aebersold and Schoville [36] implemented a live-actor bullying simulation followed by guided reflection, enabling students to explore emotional reactions and professional responses. Rose et al. [32] utilized a semi-virtual reality platform (VNurse Lite) to simulate incivility events and enhance decision-making under stress. Tsai and Chou [28] incorporated avatar-led role-play through a mobile application, integrating cognitive rehearsal within interactive scenarios. Additionally, Razzi and Bianchi [40] included simulation and scripted role-play as part of a multi-phase cognitive rehearsal training to improve nurses’ communication and confidence.

#### 3.4.4. Team-Based and Institutional Interventions (Three Studies)

System-level and team-oriented interventions were described in three studies, all aimed at cultivating a culture of civility within healthcare or academic environments. Garcia et al. [34] implemented a large-scale, multi-unit civility program across a pediatric health system using the *Plan-Do-Study-Act model*, resulting in reduced incivility and turnover. Tecza et al. [16] assessed nursing students’ perceptions of incivility during clinical placements and introduced targeted, unit-level interventions based on survey findings, which led to improved perceptions of civility in the clinical learning environment. Lasater et al. [43] delivered a six-month, three-phase civility initiative targeting student–preceptor relationships and leadership practices.

#### 3.4.5. Feedback and Communication Strategies (Two Studies)

Two studies highlighted the role of communication and feedback training in managing incivility in nursing education and practice. Altmiller et al. [41] implemented multisite strategies that emphasized constructive feedback, leading to improved communication skills and increased confidence in addressing incivility. Nikstaitis and Simko [37] delivered a case-based intervention for ICU nurses, which enhanced awareness of uncivil behaviors and encouraged reflective communication practices.

### 3.5. Outcomes of Interventions

The 17 included studies evaluated intervention outcomes using a variety of quantitative and qualitative methods. Validated instruments such as the Nursing Incivility Scale, Negative Acts Questionnaire–Revised (NAQ-R), Uncivil Behavior in Clinical Nursing Education (UBCNE), and the Workplace Civility Index were commonly used. Additional self-report tools captured outcomes related to confidence, emotional distress, communication effectiveness, and readiness to respond to incivility.

Across the studies, several reported statistically significant reductions in perceived or self-reported incivility following intervention implementation. For example, Abedini et al. [44] and Razzi and Bianchi [40] documented post-intervention decreases in incivility scores. Garcia et al. [34] reported significant reductions in uncivil workplace behaviors and voluntary turnover among registered nurses following the implementation of a system-wide civility initiative. Tecza et al. [16] and Lasater et al. [42] noted improvements in students’ perceptions of the clinical environment after implementing targeted or multi-phase civility programs. Multiple studies reported increased self-efficacy, communication confidence, and preparedness to address incivility, particularly those involving cognitive rehearsal and simulation-based strategies [24,29,33,41,43].

**Table 1:** Study characteristics.

Country/Author/Year	Purpose	Design/Methods	Participants/Settings	Intervention	Analysis	Key Findings/Outcomes
United States/Kim et al. (2024) [24]	The aim of this study was to evaluate the effectiveness of an interactive program designed to reduce nursing students’ perceived stress and improve self-efficacy and readiness to professionally address incivility during clinical practice.	Mixed-methods study, experimental pre–post-intervention design, Uncivil Behavior in Clinical Nursing Education (UBCNE; 12 items), Perceived Stress Scale (PSS; 10 items), General Self-Efficacy Scale (GSE; 10 items), and a sample characteristics questionnaire (11 items).	35 senior BSN students from a California State University; focus group (n = 11) in Spring 2024.	A 3-week interactive clinical incivility management program (1 h/week): included videos, discussions, education, and role-play using the DESC framework.	Quantitative: used paired sample *t*-tests and Pearson correlation to compare pre- and post-intervention scores on incivility (UBCNE), stress (PSS), self-efficacy (GSE), and readiness to respond. Qualitative: thematic analysis using Colaizzi’s method from a 1-h focus group with 11 students.	-Approximately 71.4% of students experienced clinical incivility, mostly from nurses. -No significant changes in stress or self-efficacy scores. -Significant improvement in students’ professional readiness to respond to incivility (*p* < 0.001). -Positive correlation found between incivility and stress levels. -Themes identified: uncivil behaviors from nurses; emotional discouragement; lack of clinical teaching support; need for formal interventions to manage incivility.
United States/Clark et al. (2023) [43]	To examine the effects of cognitive rehearsal (CR) training on newly graduated nurses’ ability to handle workplace incivility (WI) and its physiological, psychological, and patient care impacts.	Mixed-methods design of quasi-experimental study using a three-group simulation model with biometric data collection (e.g., heart rate, salivary alpha amylase), pre- and post-questionnaires (resilience and stress), and qualitative debriefings.	11 newly graduated nurses (<6 months post-graduation) from a Western U.S. state; university-based simulation setting.	A 60-min CR workshop using the TeamSTEPPS CUS model, scenario-based role-play, and debriefing. Groups experienced either a hurried (non-uncivil), uncivil, or uncivil handoff with post-intervention CR.	Quantitative data: mixed between–within subjects’ analysis of variance (ANOVA). Qualitative data: debriefing session transcripts.	-No statistically significant quantitative results due to small sample size, but trends showed decrease resilience and increase stress in uncivil handoff groups. -Qualitative data indicated participants felt unprepared despite CR training; WI disrupted communication and compromised patient care. -More time and repeated practice are needed for effective CR application.
South Korea/Kim et al. (2023) [8]	To examine the effect of a clinical incivility management module on nursing students’ perceived stress, self-efficacy, and preparedness to respond to incivility.	Quasi-experimental post-test-only non-equivalent comparison group design; used both quantitative and qualitative data.	187 senior BSN students from a nursing college in Seoul, South Korea; 94 in the control group, 93 in the experimental group.	A 2-h interactive clinical incivility management module that included video, lectures, small group discussions, scenario-based learning, and debriefing.	Quantitative: Mann–Whitney U test, Spearman correlation. Qualitative: thematic analysis of debriefing session notes.	-Approximately 72.7% reported experiencing clinical incivility. -Experimental group reported significantly lower incivility scores (*p* < 0.001). -No significant change in stress or self-efficacy. -Preparedness to respond to incivility significantly improved (*p* = 0.004). -Themes included increased awareness, need for more time, and shared experiences of incivility.
Taiwan/Tsai and Chou (2023) [38]	To develop and evaluate a smartphone application (“Easy Play Communication”) combining cognitive rehearsal and simulation to help nurses manage workplace incivility and bullying.	Two-phase study using the Analysis Design Development Implementation Evaluation (ADDIE) instructional design model. Phase 1: app development with expert feedback and user needs analysis. Phase 2: single-group pre-test–pos-ttest design.	Phase 1: 41 nurses for needs assessment; Phase 2: 47 nurses recruited online (27 completed post-test). Participants were hospital nurses in Taiwan.	A smartphone app offering training in three parts: education on incivility/bullying, interactive video scenarios, and role-play dialogue practice using Android voice input.	Descriptive statistics and paired sample *t*-tests using SPSS; evaluated changes in NAQ-R (Negative Acts Questionnaire–Revised; bullying) and Nursing Incivility Scale scores from pre-test to post-test.	-High user satisfaction (over 88% across categories). -No significant reduction in incivility or bullying scores, but qualitative feedback showed increased awareness and engagement. -Peers were the most frequent source of incivility/bullying. -The app was seen as accessible, practical, and relevant.
Iran/Kousha et al. (2022) [39]	To investigate the effectiveness of educational intervention and cognitive rehearsal on perceived incivility among emergency nurses.	Randomized controlled trial (RCT) with parallel groups and single blinding. Conducted from December 2019 to March 2020.	80 emergency nurses (40 in intervention group from Hospital A, 40 in control group from Hospital B) working in emergency departments of two public hospitals affiliated with Shahid Beheshti University of Medical Sciences, Tehran, Iran.	Intervention group received a cognitive rehearsal program over five two-hour sessions across three weeks, including definitions of incivility, ten common uncivil scenarios, appropriate responses, and role-playing. Control group received only written information about incivility.	Statistical tests (*t*-tests and analysis of variance) were used to compare incivility scores before and after the intervention, adjusting for age and work experience.	-The cognitive rehearsal training did not reduce incivility. -Incivility scores increased in the intervention group and slightly decreased in the control group. -No significant changes were found in incivility from patients, physicians, or peers. -Findings suggest increased awareness may have led to higher reporting of incivility. -Organizational factors may limit the short-term effectiveness of individual-level training.
Iran/Abedini et al. (2021) [44]	To determine the effect of student manner’s training on the uncivil behavior of nursing students in Qom University of Medical Sciences.	Quasi-experimental study with intervention and control groups. Pre- and post-intervention surveys using the Incivility in Nursing Education questionnaire (INE-R) by Clark.	83 second- and third-year nursing students from Qom University of Medical Sciences: 44 students in the experimental group, 39 in the control group.	A 4-week training program on student etiquette, delivered via online resources and in-person free-thinking sessions (2 h each, four sessions total), facilitated by Islamic education faculty.	Independent and paired *t*-tests were used to compare incivility scores before and after the intervention.	The experimental group showed a significant reduction in incivility scores after the training (from 45.37 to 39.23, *p* < 0.05), while the control group showed no significant change. Dialogue-based, reflective learning increased students’ awareness and promoted more respectful behavior.
United States/Garcia et al. (2021) [34]	To implement and evaluate a civility program aimed at reducing workplace incivility and improving teamwork, safety, and staff satisfaction across a pediatric healthcare system.	Pre–post-intervention experimental study; quality improvement project using the Plan-Do-Study-Act model; included pre- and post-program surveys and education sessions.	Over 1500 interprofessional staff (including nurses, physicians, therapists, and other staff) across 20 clinical areas in a large pediatric teaching hospital in Texas. Pre-program survey: 209 staff; post-program survey: 223 staff.	Civility education delivered through online modules and in-person Clinical Nurse Specialist (CNS)-led training sessions. Included unit-specific uncivil scenarios, self-assessments, and action planning. Classes emphasized respectful communication and reporting processes.	Pre- and post-intervention comparisons using Negative Acts Questionnaire–Revised (NAQ-R) survey scores. Civility Quotient used for self-reflection. Trends in turnover and incident reports were monitored.	-Significant reduction in reported uncivil behaviors across all NAQ-R items. -Voluntary turnover rate decreased from 13.2% to 10.9%. -Participants reported greater willingness to speak up, reflect on behavior, and use conflict resolution strategies. -High participation rates and system-wide adoption indicated growing support and positive culture change.
United States/McDermott et al. (2021) [33]	To evaluate the effectiveness of cognitive rehearsal training in equipping newly licensed registered nurses (NLRNs) with strategies to recognize and respond to workplace incivility.	Mixed-methods design for program evaluation; quantitative: a five-item web-based survey administered three months post-intervention to assess the frequency of witnessed and addressed incivility; qualitative: open-ended survey responses analyzed using inductive content analysis to explore participants’ experiences and perceptions.	114 newly licensed registered nurses (RNs) enrolled in nurse residency programs (NRPs) responded to the post-intervention web-based survey, providing both quantitative and qualitative data.	A one-hour educational session on workplace incivility, using cognitive rehearsal, was delivered as part of a nurse residency program. The session included didactic instruction, scripted role-play, cue cards, and practice opportunities to personalize responses to uncivil behaviors.	Quantitative data were analyzed using descriptive statistics from a five-item post-intervention survey. Qualitative responses to an open-ended question were examined using inductive content analysis to identify recurring themes.	Of 114 nurse residents, 55% witnessed incivility, and 45% responded. Most found the training helpful (56%), especially those who witnessed incivility (65.6%), though only 36% valued the cue cards. Qualitative themes highlighted the need for improved scripting and broader leadership engagement to address systemic incivility.
United States/Johnson et al. (2020) [45]	To investigate how exposure to incivility affects clinical performance, teamwork, and emotional state during a simulation-based cardiopulmonary resuscitation scenario.	Randomized controlled trial (RCT) in a simulation lab; experimental group exposed to incivility; control group received a neutral interaction. Both groups completed a CPR simulation task and post-simulation assessments.	58 registered nurses enrolled in a Bachelor of Science in Nursing completion program, randomized into teams (2–4 members per team). Conducted at a university-based simulation center.	A brief simulated episode of incivility delivered by a lab actor before a clinical CPR scenario. Control group received a neutral greeting.	(a) Descriptive statistics for survey responses. (b) Comparison of pre- and post-program NAQ-R survey results. (c) Analysis of voluntary turnover rates before and after program implementation.	-No significant differences in overall CPR, teamwork, or cognitive scores between groups. -However, 66% of experimental teams made a major error (administering two shocks) vs. 0% in control teams, violating CPR protocol. -Emotional states changed in both groups, but only hostility showed a statistically significant difference (*p* = 0.045). -Findings suggest even brief exposure to incivility may contribute to serious clinical errors.
United States/Rose et al. (2020) [32]	To determine if a semi-virtual reality simulation could improve nursing students’ awareness of civility and incivility in themselves and others.	Pre–post-test experimental design. Intervention group used a web-based simulation tool (VNurse Lite) and attended a debriefing session; control group received only a traditional lecture.	53 senior nursing students from a private nursing program in the Midwest U.S.; 27 in the intervention group and 26 in the control group.	Semi-virtual reality simulation (VNurse Lite app) where students interacted with avatars to observe and practice civil behaviors, followed by a debriefing based on social cognitive theory.	Analysis of covariance, chi-square tests, and content analysis for open-ended responses. The Nurses’ Intervention for Civility in Education Questionnaire was used to measure outcomes; Pearson correlation.	-No significant difference in overall civility scores between groups, but intervention group had higher awareness of academic incivility (*p* = 0.005). -Qualitative data revealed increased self-awareness and commitment to intervene in uncivil situations. -Themes included awareness of others’ incivility, personal accountability, and readiness to act.
United States/Howard and Embree (2020) [35]	To evaluate the effectiveness of an asynchronous e-learning intervention on improving nurses’ communication skills and awareness in managing workplace incivility.	Mixed-methods (quasi-experimental design of pre-test–post-test using the Workplace Civility Index). Both quantitative and qualitative data were collected.	49 nurses at an academic medical center in the Midwestern United States (21 in the experimental group, 28 in the control group). Participants were mostly early-career nurses across ICU, emergency, and medical–surgical units.	A 2.5 h asynchronous e-learning course, “Bullying in the Workplace: Solutions for Nursing Practice”, developed with Sigma. Included interactive modules with branching scenarios to practice communication strategies.	Descriptive statistics and paired sample *t*-tests. The Workplace Civility Index was used to assess civility levels pre- and post-intervention.	-Civility scores improved significantly in the experimental group (from 91.6 to 95.4, *p* < 0.00001). -Civility scores decreased in the control group (from 88.2 to 80.2, *p* = 0.0002). -All experimental group participants reported successfully using a positive conflict management strategy. -The program increased communication confidence and civility awareness.
United States/Aebersold and Schoville (2020) [36]	To explore how a simulated bullying experience affects senior nursing students’ understanding of bullying, emotional responses, and strategies to manage incivility in clinical settings.	Qualitative study using post-simulation reflection surveys.	169 senior Bachelor of Science in Nursing students in a Leadership and Management course at a Midwestern U.S. nursing school. Simulation took place in a clinical learning lab.	A 2 h bullying simulation with an embedded actor playing a bully nurse, followed by a structured debriefing. Students prepared with assigned readings and a knowledge quiz.	Qualitative thematic analysis based on Graneheim and Lundman’s approach. Reflections were coded and analyzed to identify recurring themes.	-Six major themes emerged: (a) chaotic environment, (b) great learning experience, (c) emotional response, (d) bullying behaviors, (e) barriers to learning, and (f) impact. -Students described the simulation as emotionally intense but educationally valuable. -The experience increased their awareness of bullying and helped them practice and reflect on strategies for responding to incivility in clinical practice. -Debriefing was essential in helping students process their feelings and apply learning to real-world situations.
United States/Razzi and Bianchi (2019) [40]	To implement and evaluate a quality improvement program using cognitive rehearsal training to reduce incivility among nurses.	A one-group pre-test–post-test experimental design with repeated measures at three time points was used as part of a quality improvement project.	24 registered nurses from a 232-bed community hospital in the Northeastern United States. Participants worked across units and roles, including administration and care management.	A 1 h educational session and cognitive rehearsal training, including scripted responses, role-playing, and practice in identifying and addressing incivility. Participants completed the Nursing Incivility Scale before, immediately after, and one month after the intervention.	One-way repeated-measures analysis of variance to assess total and subscale scores of the Nursing Incivility Scale. Descriptive statistics used for demographics and post-program evaluations.	-Significant reductions in overall incivility scores and five out of eight subscales (inappropriate jokes, gossip/rumors, free riding, abusive supervision, lack of respect). -All eight subscales showed decreased mean scores post-intervention. -Participants reported high satisfaction with the training and greater confidence in addressing incivility. -Authors recommend broader implementation and a formal incivility policy to sustain improvements.
United States/Altmiller et al. (2018) [41]	To explore undergraduate nursing students’ perceptions of giving and receiving constructive feedback after participating in a teaching strategy designed to promote feedback as a tool for professional development.	Qualitative study using Colaizzi’s method of phenomenological reduction to analyze written student reflections.	523 undergraduate nursing students from nine nursing programs across the U.S. who completed 985 discussion board posts or essays in response to the “Giving and Receiving Constructive Feedback” teaching strategy.	An 18-min narrated presentation teaching the knowledge, skills, and attitudes for giving and receiving feedback, integrated into coursework at different program levels.	Thematic qualitative analysis using Colaizzi’s approach; data were coded, clustered into themes, validated, and synthesized across all participating sites.	-Seven key themes emerged: (1) opportunity for improvement, (2) learned skill for the giver, (3) communication is essential to teamwork, (4) improving patient safety, (5) reframing negative emotional responses, (6) self-reflection is a key component, and (7) need to be open to feedback. -Students acknowledged the emotional challenges of receiving feedback but reported a shift toward viewing it as constructive and essential for growth. -The strategy fostered self-awareness, improved communication, and reinforced the role of feedback in safe and effective nursing practice.
United States/Tecza et al. (2018) [16]	To measure nursing students’ perceptions of incivility in the clinical learning environment and test the effectiveness of hospital- and unit-level interventions. A secondary aim was to evaluate the reliability of the Nursing Student Perception of Civil and Uncivil Behaviors tool.	Quasi-experimental, non-equivalent pre-test–post-test design conducted at a single pediatric hospital.	Pre-intervention: 314 nursing students out of 652 eligible students; post-intervention: 410 out of 591 students. Clinical learning experiences were conducted across 10 inpatient units of a magnet-designated pediatric hospital in the Midwest.	Hospital-wide education and unit-specific strategies, including videos, welcome cards, informational posters, and interactive exercises, were developed and implemented based on survey results.	Independent sample *t*-tests to compare pre- and post-intervention means for 12 survey items and three core constructs. Reliability tested using Cronbach’s alpha.	-Students reported a statistically significant improvement in overall civility, feeling more respected and included in their clinical learning environment. -The Nursing Student Perception of Civil and Uncivil Behaviors tool demonstrated high reliability (Cronbach’s alpha = 0.927). -Unit-specific interventions were most effective when nurse leadership was actively engaged
United States/Lasater et al. (2015) [42]	To evaluate whether a three-part educational intervention could reduce perceived incivility and improve self-efficacy and collective efficacy among nursing staff in two hospital units.	Mixed-methods study with multiple post-assessment time points over 24 months. Quantitative data were collected at six intervals; qualitative interviews followed. No control group was used.	94 participants: 63 from Unit A and 31 from Unit B of a large academic health sciences hospital in Oregon. Included registered nurses, support staff, and leadership.	A three-part program delivered over 6 months: (1) didactic session on incivility and its effects, (2) workshop with role-play and toolkits to address unit-specific concerns, (3) simulation sessions with debriefing for charge nurses and leaders.	Linear mixed-effects models used to assess changes over time in Nurse Incivility Scale, New General Self-Efficacy Scale, and Workplace Collective Efficacy Scale scores. Qualitative data explained quantitative trends.	-Perceived incivility significantly decreased over time in both units.
United States/Nikstaitis and Simko (2014) [37]	To determine if an educational intervention using case studies and discussion could increase awareness and reduce perceived incidences of incivility among ICU nurses.	Quantitative pilot study using a one-group pre–post-test design over 12 weeks. Surveys were administered before and after a 60-min in-service education.	ICU nurses (n = 38 eligible, n = 21 completed full study) at Saint Agnes Hospital’s Adult ICU in Baltimore, Maryland.	A 60-min educational session using case studies, literature review, and discussion on incivility and professional behavior; offered multiple times to accommodate shifts.	Descriptive statistics and *t*-tests; hierarchical regression to identify predictors of perceived incivility. NIS (Nursing Incivility Scale) used for measurement.	-No significant pre–post change in incivility scores -Perceived incivility increased post-intervention, suggesting greater awareness. -Patients/families were seen as most uncivil; supervisors perceived as most civil. -Predictors of higher incivility perception included race, >5 years in nursing, part-time status, and younger age.

Not all interventions showed consistent or statistically significant changes. For example, Tsai and Chou [38] and Kousha et al. [39] reported increased awareness of incivility but no significant change in incivility scores. Nikstaitis and Simko [37] observed a post-intervention rise in reported incivility, which the authors attributed to heightened recognition rather than a true increase in behaviors. Simulation-based studies also measured emotional and behavioral responses. Johnson et al. [45] found that nurses exposed to incivility were significantly more likely to make critical errors during cardiopulmonary resuscitation (CPR) simulations than those in the control group. Notably, 66% of participants in the incivility group committed a major protocol error, while none in the control group did. These findings suggest that incivility can undermine clinical performance and adherence to protocols in high-stress situations. Aebersold and Schoville [36] used actor-led simulations to measure stress response and participant reflection. Rose et al. [32] found that a semi-virtual simulation platform improved recognition of incivility and participant confidence in responding.

Qualitative data from several studies highlighted changes in students’ self-awareness, emotional reactions, and communication skills. Participants reported greater recognition of unprofessional behaviors, as well as improvements in managing their own responses. Themes of shared experience, peer support, and leadership engagement emerged as consistent, contextual factors influencing intervention outcomes [29,32,34,42].

### 3.6. Approach to Risk of Bias Assessment

An informal risk of bias assessment was conducted for all 17 included studies based on five key methodological domains: study design, sample size, intervention clarity, outcome measurement, and presence of a control group. Among the studies, three—Tecza et al. [16], Garcia et al. [34], and Kousha et al. [39]—were rated as having a low risk of bias due to their use of validated instruments, adequate sample sizes, and either randomized or system-wide intervention designs with measurable outcomes. Thirteen studies were assessed as having a moderate risk of bias, including Kim et al. [24], Kim et al. [29], Rose et al. [32], McDermott et al. [33], Howard and Embree [35], Aebersold and Schoville [36], Tsai and Chou [38], Razzi and Bianchi [40], Altmiller et al. [41], Lasater et al. [42], Clark et al. [43], Abedini et al. [44], and Johnson et al. [45]. These studies generally featured appropriate educational interventions and meaningful outcome measures but were limited by small sample sizes, absence of comparison groups, short-term follow-up, or reliance on self-reported data. One study, Nikstaitis and Simko [37], was rated as having a high risk of bias due to its very small sample size, lack of a control group, and minimal statistical or behavioral outcome data.

## 4. Discussion

This systematic review highlights a range of evidence-based interventions implemented over the past decade to address clinical incivility in nursing. While the Results section outlined measurable improvements across multiple outcomes, this Discussion interprets those findings through the lens of professional development, learning theory, and systems-based change. The consistent emphasis on simulation, cognitive rehearsal, and structured educational programs reflects a pedagogical shift toward experiential and reflective strategies in nursing education.

Simulation-based interventions, applied across multiple studies, are grounded in Kolb’s Experiential Learning Theory [46], which posits that effective learning occurs through concrete experiences followed by reflective observation. Of note, studies incorporating high-fidelity or virtual simulation facilitated the rehearsal of conflict resolution strategies in psychologically safe environments. These simulations not only enhanced communication skills but allowed participants to experience and regulate the emotional responses often triggered by incivility. As shown in prior research [24,36,45], simulation-based interventions may support long-term professional behavior change, particularly when paired with structured debriefing. This aligns with findings by Roberts [28], who developed the “*Inhibiting Actions Theory*” to describe how incivility undermines nursing students’ clinical learning and identity formation, highlighting the need for interventions that foster psychological safety and supportive educational environments.

Cognitive rehearsal, used in both digital and face-to-face formats, was another frequently implemented approach. Although originally developed for workplace bullying, cognitive rehearsal shows promise in addressing student incivility. Practicing scripted responses in advance appears to improve confidence and reduce the anxiety associated with confrontation. However, program effectiveness may depend on contextual factors such as organizational support, leadership [47], and psychological safety [48]. These findings reinforce the need to implement cognitive rehearsal as part of a broader, system-wide civility strategy, consistent in Givier and Varagona’s integrative review of incivility in nursing and leadership intervention [49].

Educational modules were the most used intervention type but varied in structure, intensity, and evaluation methods. While most studies utilizing educational modules reported gains in knowledge and awareness, fewer assessed behavioral outcomes or long-term retention. This mirror concerns raised in the prior literature about the risk of “one-off” training sessions producing superficial or temporary gains [3]. Programs integrating discussion, case analysis, and repeated practice—such as those described by Kim et al. [24,29]—were more likely to yield improvements in readiness and professional identity formation.

Notably, interventions that incorporated team-based strategies and leadership support reported more sustained outcomes. These system-level approaches suggest that individual training is insufficient without broader institutional alignment. For example, Garcia et al. [34] demonstrated reduced incivility and staff turnover through a quality improvement framework that included policy revision and continuous feedback. This finding is consistent with studies outside of nursing, which emphasize the role of organizational climate and leadership behavior in promoting psychological safety and civility [50].

Moreover, it is important to further address patterns and trends in simulation-based interventions and cognitive rehearsal, which incorporate communication strategies, reflection, and feedback. As one of the first concepts demonstrated in foundational theory and skills for nursing practice courses, therapeutic communication is essential in all aspects of nursing and exhibits measurable improvements discussed in this review. Incorporation of feedback strategies, such as observation and reflection through debriefing, demonstrated the greatest impact in facilitating the *lived experience* of both dealing with situations of incivility and also fostering assertiveness and improved confidence in how to handle a compromising situation and how “it feels” to confidently assert a civil response in an uncomfortable environment. Simulation-based interventions and cognitive rehearsal can be further enhanced with reenactments of situations of shared experiences of incivility with senior nursing students in both impromptu and scheduled town halls, fostering a sense of community. Providing skills “bootcamps” prior to the start of each new semester and simulations that include students from different cohorts allows for structured support and a sense of belonging in a team-based intervention.

Despite these promising trends, this review revealed several limitations in the existing literature. Many studies used small, single-site samples, lacked control groups, or relied solely on self-reported outcomes. In addition, the heterogeneity of tools used to measure incivility—ranging from validated scales to informal checklists—limited cross-study comparisons. The absence of longitudinal follow-up in most studies raises concerns about the durability of intervention effects. As also noted by Kim et al. [29], long-term monitoring is critical to assessing whether interventions produce enduring culture change or short-term behavioral adjustments.

Future research should prioritize methodological rigor and contextual sensitivity. Multisite randomized controlled trials, the use of standardized measurement tools, and the inclusion of diverse populations across geographic and cultural contexts would improve generalizability. Moreover, studies should assess not only immediate outcomes but also long-term indicators such as student retention, staff well-being, and patient safety outcomes.

Lastly, reiterating the importance of reflective practice and peer support emerged across both quantitative and qualitative findings. These elements appear to strengthen the impact of formal training by fostering emotional intelligence, professional identity, and solidarity in the face of hierarchical challenges. Fostering interventions whereby students in different cohorts come together will further enhance a more realistic model of working with levels of experience as a student. As such, and as discussed above, civility-promoting interventions should be embedded within curricula, mentorship models, and organizational development frameworks, moving beyond reactive responses to incivility and toward proactive culture-building.

## 5. Conclusions

This systematic review highlights a growing body of evidence supporting the use of structured interventions—such as cognitive rehearsal, simulation, and educational modules—to reduce clinical incivility in nursing education and practice. These interventions were generally effective in enhancing participants’ confidence, communication, and readiness to address incivility. However, methodological limitations, variability in intervention delivery, and a lack of long-term evaluation suggest a need for more rigorous and standardized research.

Future efforts should focus on sustained, culturally sensitive, and system-level strategies to cultivate psychologically safe learning and work environments. Demonstrating a culture of civility requires role models from the start of and throughout the nursing students’ academic and clinical experience, along with the use of consistently structured interventions consistently threaded in curricula and graduate residency programs. One cannot expect a reduction of incivility in nursing without clear, consistent, and safe reporting policies, along with leadership accountability and professionalism. Ultimately, promoting civility in nursing is not only essential for student success and workforce retention but is also integral to delivering high-quality, respectful, and safe person-centered patient care. It is the responsibility of every nurse in academia, clinical practice, and communities to be held accountable as an example and mentor to other nurses before and after them for the consistent practice of the art and science of nursing under the umbrella of the crucial component of civility.

## Data Availability

No new data were created or analyzed in this study. Data sharing is not applicable to this article.

## Public Involvement Statement

No public involvement in any aspect of this research.

## Guidelines and Standards Statement

The manuscript was conducted in accordance with the Preferred Reporting Items for Systematic Reviews and Meta-Analyses (PRISMA) guidelines from https://www.equator-network.org/reporting-guidelines/prisma/ (accessed on 1 December 2024).

## Use of Artificial Intelligence

Artificial intelligence (AI) tools were utilized in a limited capacity during the preparation of this manuscript. Specifically, ChatGPT 4o (developed by OpenAI) was used to assist with grammar refinement, the organization and formatting of reference citations, and the creation of figures to visually illustrate the study findings. All AI-assisted content was thoroughly reviewed and edited by the authors to ensure accuracy, clarity, and alignment with academic standards. AI tools were not used for the generation of original research findings, data analysis, or interpretation.
